# Transdifferentiation of pancreatic progenitor cells to hepatocyte-like cells is not serum-dependent when facilitated by extracellular matrix proteins

**DOI:** 10.1038/s41598-018-22596-z

**Published:** 2018-03-12

**Authors:** Francis D. Gratte, Sara Pasic, John K. Olynyk, George C. T. Yeoh, David Tosh, Deirdre R. Coombe, Janina E. E. Tirnitz-Parker

**Affiliations:** 1 School of Pharmacy and Biomedical Sciences, Curtin Health Innovation Research Institute, Curtin University, Bentley, WA Australia; 2 School of Veterinary and Life Sciences, Murdoch University, Murdoch, WA Australia; 3 Department of Gastroenterology and Hepatology, Fiona Stanley and Fremantle Hospitals, Perth, WA Australia; 4 Cancer and Cell Biology Division, The Harry Perkins Institute of Medical Research, Nedlands, WA Australia; 5 Centre for Regenerative Medicine, University of Bath, Bath, United Kingdom; 6 Centre for Cell Therapy and Regenerative Medicine, School of Biomedical Sciences, The University of Western Australia, Crawley, WA Australia; 7 School of Medicine and Pharmacology, University of Western Australia, Fremantle, WA Australia

## Abstract

The rising prevalence of chronic liver disease, coupled with a permanent shortage of organs for liver transplantation, has sparked enormous interest in alternative treatment strategies. Previous protocols to generate hepatocyte-like cells (HLCs) via pancreas-to-liver transdifferentiation have utilised fetal bovine serum, introducing unknown variables and severely limiting study reproducibility. Therefore, the main goal of this study was to develop a protocol for transdifferentiation of pancreatic progenitor cells to HLCs in a chemically defined, serum-free culture medium. The clonal pancreatic progenitor cell line AR42J-B13 was cultured in basal growth medium on uncoated plastic culture dishes in the absence or presence of Dexamethasone on uncoated, laminin- or fibronectin-coated culture substrata, with or without serum supplementation. The hepatocytic differentiation potential was evaluated: (i) morphologically through bright-field and scanning electron microscopy, (ii) by assessing pancreatic and hepatic marker expression and (iii) by determining the function of HLCs through their ability to synthesise glycogen or take up and release indocyanine green. Here we demonstrate for the first time that transdifferentiation of pancreatic cells to HLCs is not dependent on serum. These results will assist in converting current differentiation protocols into procedures that are compliant with clinical use in future cell-based therapies to treat liver-related metabolic disorders.

## Introduction

Irrespective of the underlying aetiology, chronic liver diseases such as alcoholic liver disease, non-alcoholic steatohepatitis or viral hepatitis infection may progress to cirrhosis and eventually hepatocellular carcinoma. Despite a recent decline in death rates from other cancers, the prevalence and disease burden of hepatocellular carcinoma continue to rise due to an increase in risk factors such as diabetes, obesity or dietary aflotoxin B1 exposure^1,2^. Currently, tumour resection, ablation or orthotopic liver transplantation are the primary treatment options. However, the demand for donor livers drastically exceeds their availability and an increasing number of patients with end-stage liver disease die on the waiting list for transplantation, highlighting the need for alternative treatment approaches. Extracorporeal or bioartificial liver devices and hepatocyte transplantation represent two promising strategies to support the failing liver, and may either buy time for the native liver to recover through repair and regeneration, or may prolong a patient’s life until liver transplantation. For these therapies, hepatocytes can be isolated from surplus or rejected donor livers, however availabilities are restricted and the viability of the generated hepatocyte population is often compromised if harvested from livers obtained from non-heart beating cadavers^3^. As an alternative, hepatocyte-like cells (HLCs) have been generated, with variable rates of efficacy, from a variety of cell sources including embryonic stem cells, mesenchymal stem cells, induced pluripotent stem cells and human amniotic stem cells^4^.

Pancreatic progenitor cells have also been studied as hepatocyte precursors. In particular the pancreatic progenitor cell line AR42J-B13 has been used as a pancreas-to-liver transdifferentiation model^5–8^. Transdifferentiation belongs to a wider class of cell transformations termed metaplasias and refers to the phenomenon of one differentiated cell type irreversibly converting to another^9^. A natural case of metaplasia is the development of Barrett’s metaplasia in the context of severe gastroesophageal reflux disease, where normal stratified squamous epithelial cells in the distal oesophagus are replaced by a simple columnar epithelium that includes acid mucin-containing goblet cells - a cell type normally found in the gastrointestinal tract^9^. Pancreas-to-liver transdifferentiation reflects the close developmental relationship of the two tissues, both of which arise from the same endodermal region during embryogenesis^10^. Pancreas-derived HLCs can be induced *in vivo* by subjecting rats to a copper depletion-repletion protocol^11^ or by transgenically overexpressing keratinocyte growth factor in pancreatic β-cells^12^. *In vitro*, pancreas-to-liver transdifferentiation can be successfully recapitulated with the amphicrine cell line AR42J, which was derived from azaserine-treated rats^13^. Treatment of the subclone AR42J-B13 with the synthetic glucocorticoid Dexamethasone induces these cells to convert to a functional hepatocytic phenotype, as determined by downregulation of amylase with concomitant induction of liver-enriched transcription factors and the expression and activity of hepatocyte-specific proteins^5,14^.

These previous studies have been performed in culture medium containing fetal bovine serum (FBS) to supply a cocktail of the nutrients, growth factors, hormones, carrier proteins for lipoid substances and trace elements that are necessary for substratum attachment, cell viability and proliferation. However, ethical concerns have been raised with respect to the harvest and collection of FBS, and there is considerable batch-to-batch variation in FBS, resulting in qualitative and quantitative differences in culture medium compositions. In addition, FBS may contain adverse factors such as endotoxins or haemoglobin and may be a source of bacteria, viruses and other microbial contaminants^15^. To establish reproducible differentiation techniques and to generate protocols that are potentially suitable for human cell therapy, it is desirable to create defined, serum-free cell culture conditions. Most serum-free culture approaches have used extracellular matrix (ECM) proteins to mediate cell adhesion. The ECM is comprised of various macromolecules, including glycoproteins such as collagens, laminins, fibronectin and perlecan, which together assemble into a scaffold that directs the spatial arrangements of cells and dictates tissue structures. The interaction of cells with ECM molecules via the cells’ integrin receptors triggers intracellular signalling pathways that influence cell morphology and behaviour and provide essential intracellular signals to prevent unregulated apoptosis^16,17^. The ECM components collagen type I, fibronectin and laminin 111 have been demonstrated to regulate hepatocytic differentiation and maintenance of a mature phenotype *in vitro*^18,19^. Collagen I promotes the adhesion and differentiation of liver progenitor cells into hepatocytes or cholangiocytes^17,20^ while laminin 111 was shown to increase liver progenitor cell attachment and proliferation while inhibiting differentiation and maintaining an immature phenotype^21–23^. Fibronectin generally promotes cell adhesion and in some cases differentiation, mediated via particular cell surface integrins. A study with human liver progenitors found that fibronectin promoted rapid terminal differentiation and possibly apoptosis of these cells^20,23^.

The aims of the present study were to generate functional HLCs through the transdifferentiation of pancreatic AR42J-B13 cells using a chemically defined, serum-free cell culture protocol and to evaluate and compare the traditional (with FBS) to the serum-free cultures. Accordingly, AR42J-B13 cells were grown on uncoated plastic culture dishes using basal cell culture medium containing FBS, or under transdifferentiation-inducing conditions with or without serum supplementation on culture dishes that were uncoated, laminin-coated, or fibronectin-coated. Over a period of five days, the cells were monitored and morphologically assessed, evaluated for changes in gene and protein expression of pancreatic and hepatocytic markers, as well as being subjected to functional hepatocyte assays. AR42J-B13 cells rapidly changed their morphology and their gene and protein expression patterns from a pancreatic to a hepatocytic phenotype. They also demonstrated hepatocyte functionality independent of the culture medium’s FBS status. To the best of our knowledge, this is the first report of successful hepatocytic transdifferentiation of pancreatic cells in serum-free conditions, facilitated by ECM proteins.

## Results

### Morphological changes in AR42J-B13 cells

Possible changes in AR42J-B13 cell morphology were investigated under the following conditions: (1) undifferentiated cells grown under basal culture conditions (undiff); all subsequent conditions used differentiation medium and were: (2) cells grown on tissue culture plastic without ECM coating in FBS-containing medium (differentiation control group), cells grown on fibronectin with (3), and without FBS (4), and cells grown on laminin with (5), and without FBS (6). Cells were also cultured on tissue culture plastic without an ECM coating or serum supplementation but these cells were not viable (results not shown) and were excluded from further analyses.

**Figure 1:**
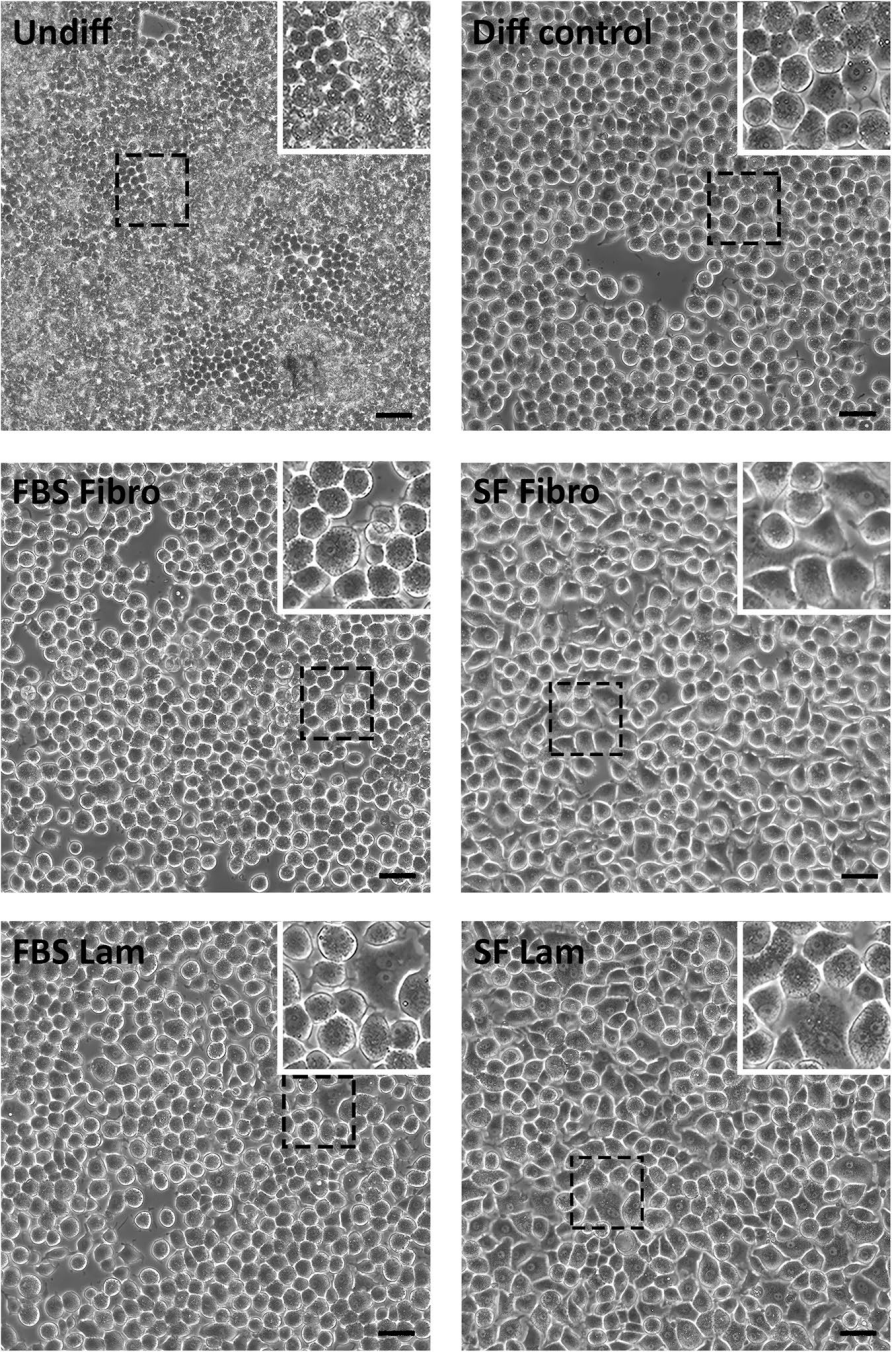
Morphology of undifferentiated AR42J-B13 cells and cells subjected to differentiation-inducing conditions, assessed by phase contrast microscopy on day 4. Undiff - undifferentiated cells; Diff control - differentiation control, with FBS, on plastic; FBS Fibro - differentiation medium, with FBS, on fibronectin; SF Fibro - differentiation medium, serum-free, on fibronectin; FBS Lam - differentiation medium, with FBS, on laminin; SF Lam - differentiation medium, serum-free, on laminin. Scale bar = 50 µm.

**Figure 2:**
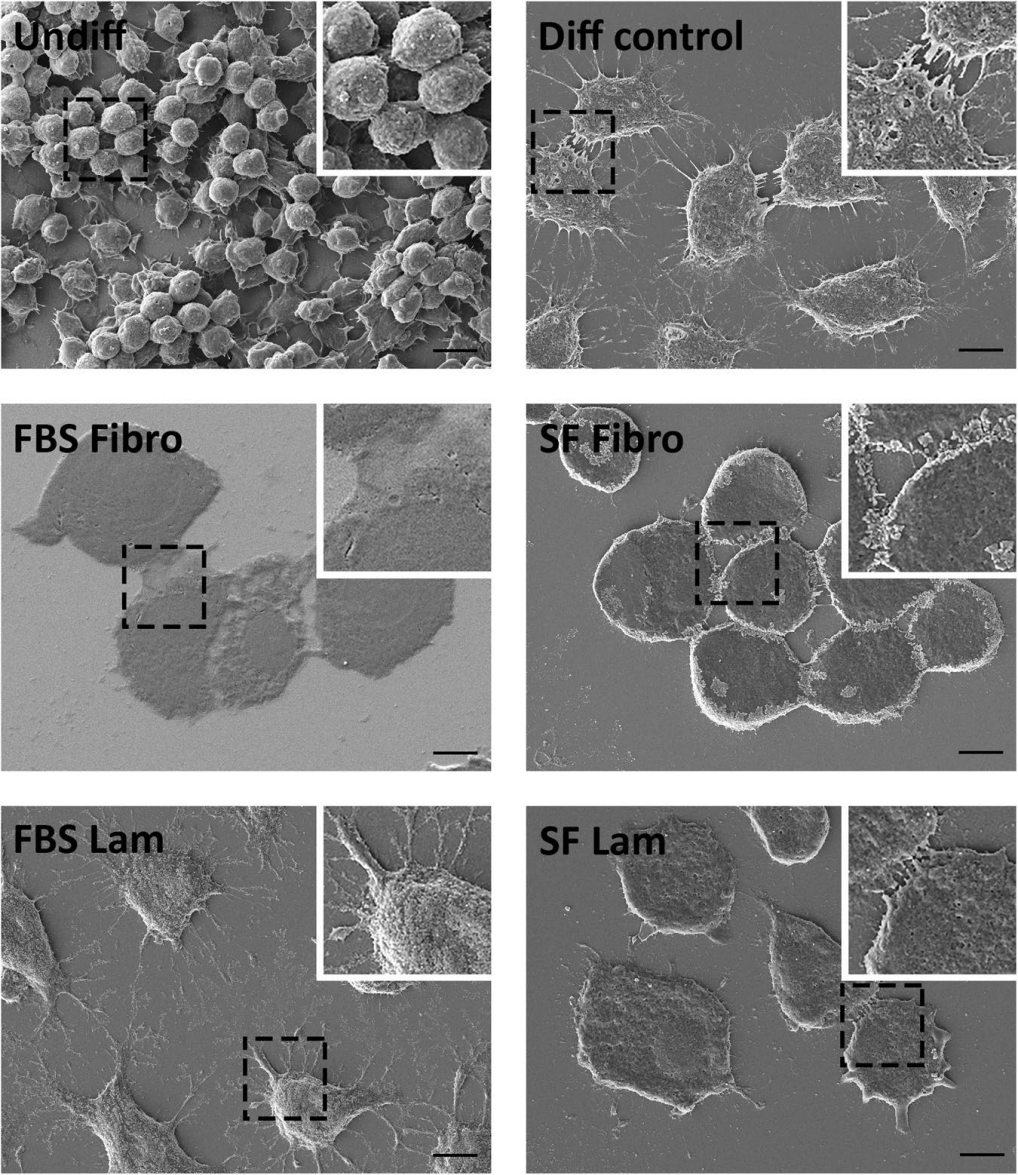
Morphology of undifferentiated AR42J-B13 cells and cells subjected to differentiation-inducing conditions, assessed by scanning electron microscopy on day 5. Undiff - undifferentiated cells; Diff control - differentiation control, with FBS, on plastic; FBS Fibro - differentiation medium, with FBS, on fibronectin; SF Fibro - differentiation medium, serum-free, on fibronectin; FBS Lam - differentiation medium, with FBS, on laminin; SF Lam - differentiation medium, serum-free, on laminin. Scale bar = 10 µm.

Undifferentiated, proliferating AR42J-B13 cells in basal culture medium displayed a small (~10 µm), round morphology and grew in clusters (Fig. 1, top left). Upon exposure to transdifferentiation-inducing conditions, they quickly (by day 2 in differentiation medium) enlarged to cells of 20–40 µm in diameter, became less spherical, flattened and formed monolayers of mostly polygonal cells. By day 4 of the protocol, AR42J-B13 cells had prominent nuclei and in some cases exhibited multinucleation, a common characteristic seen in 40–50% of healthy, adult hepatocytes. These rapid and extensive morphological changes were observed in all experimental groups subjected to differentiation medium, regardless of the choice of the ECM protein or the availability of FBS (Fig. 1). Scanning electron microscopy of AR42J-B13 cells on day 5 post-induction of differentiation confirmed that all cells exposed to differentiation medium underwent these morphological changes, including cell enlargement and flattening, irrespective of whether they were grown on plastic with FBS or on ECM-coated dishes, with or without serum supplementation (Fig. 2). Live cell imaging of cells grown under transdifferentiation-inducing conditions on laminin and without FBS for four days further illustrated how quickly and effectively these conversions were induced with clear evidence of differentiation two days after induction (Supplemental Video 1).

### Transcriptomic changes of AR42J-B13 cells exposed to differentiation medium

**Figure 3:**
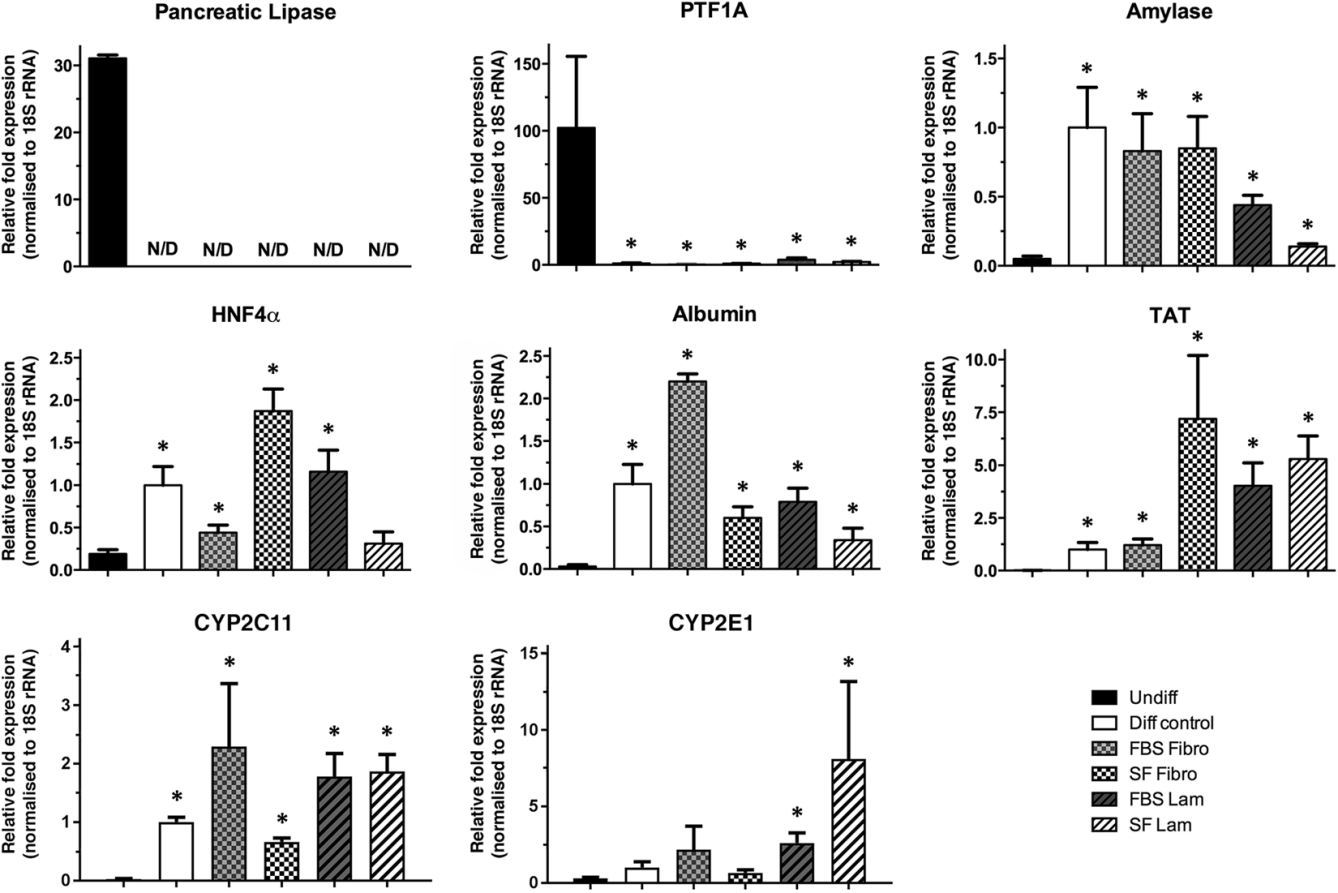
Gene expression of pancreatic (pancreatic lipase, pancreas-specific transcription factor 1a, amylase) and hepatocytic markers (hepatocyte nuclear factor 4 α, albumin, tyrosine aminotransferase, cytochrome P450 2C11 and 2E1) in undifferentiated AR42J-B13 cells and cells subjected to differentiation-inducing conditions on day 5. The data are expressed as means ± SEM with *n* = 3–5 and *p < 0.05 compared with undifferentiated cells. Undiff - undifferentiated cells; Diff control - differentiation control, with FBS, on plastic; FBS Fibro - differentiation medium, with FBS, on fibronectin; SF Fibro - differentiation medium, serum-free, on fibronectin; FBS Lam - differentiation medium, with FBS, on laminin; SF Lam - differentiation medium, serum-free, on laminin.

To evaluate the switch from a pancreas to a hepatocyte phenotype at the molecular level, mRNA expression levels of pancreatic and hepatocytic marker proteins as well as cytochrome P450 proteins were assessed on day 5 of differentiation. Expression levels of the pancreatic enzyme amylase were significantly elevated at this time point, consistent with Dexamethasone-induced transient increases shown previously^5^. However, expression of pancreas-specific PTF1A, a master regulator of pancreas development and acinar cell fate specification, which is highly expressed in normal AR42J-B13 cells, was significantly reduced to almost undetectable levels in all experimental groups exposed to differentiation medium. Additionally, the clinically relevant enzyme pancreatic lipase was downregulated and no longer measurable, a clear difference from the expression seen in control cells at day 5. In contrast, expression levels of the hepatocyte markers HNF4α, albumin, TAT and cytochrome P450 enzymes CYP2C11 and CYP2E1 were all upregulated to varying degrees in response to differentiation medium, indicating that the pancreatic AR42J-B13 cells had undergone a conversion towards a hepatocyte phenotype within this short time frame (Fig. 3).

### Transdifferentiated AR42J-B13 express hepatocyte proteins

**Figure 4:**
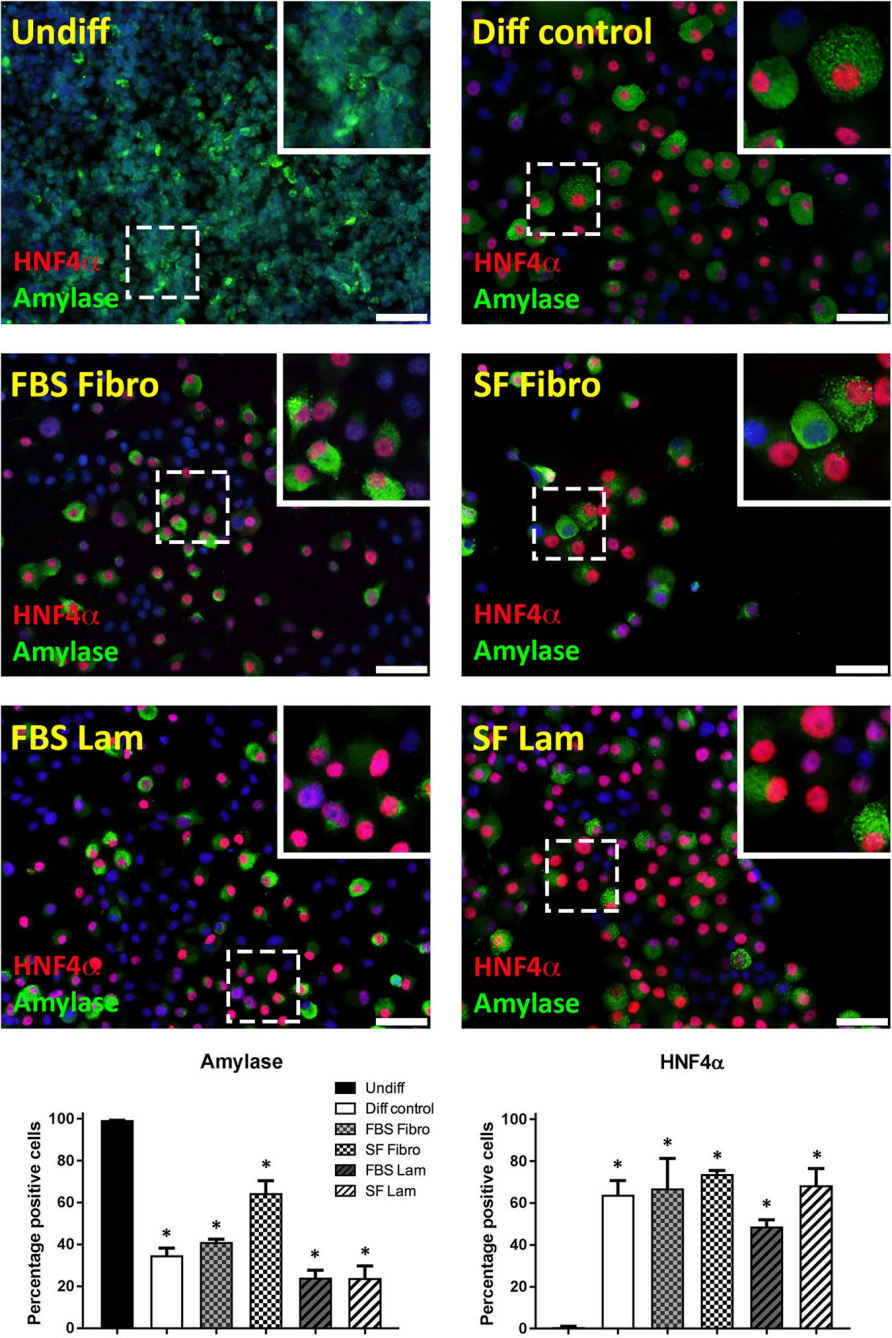
Immunofluorescent staining and quantification of pancreatic amylase (green) and hepatocyte nuclear factor 4 α (green) in undifferentiated AR42J-B13 cells and cells subjected to differentiation-inducing conditions on day 5, with DAPI (blue) for nuclear quantitation. Undiff - undifferentiated cells; Diff control - differentiation control, with FBS, on plastic; FBS Fibro - differentiation medium, with FBS, on fibronectin; SF Fibro - differentiation medium, serum-free, on fibronectin; FBS Lam - differentiation medium, with FBS, on laminin; SF Lam - differentiation medium, serum-free, on laminin. Scale bar = 50 µm.

**Figure 5:**
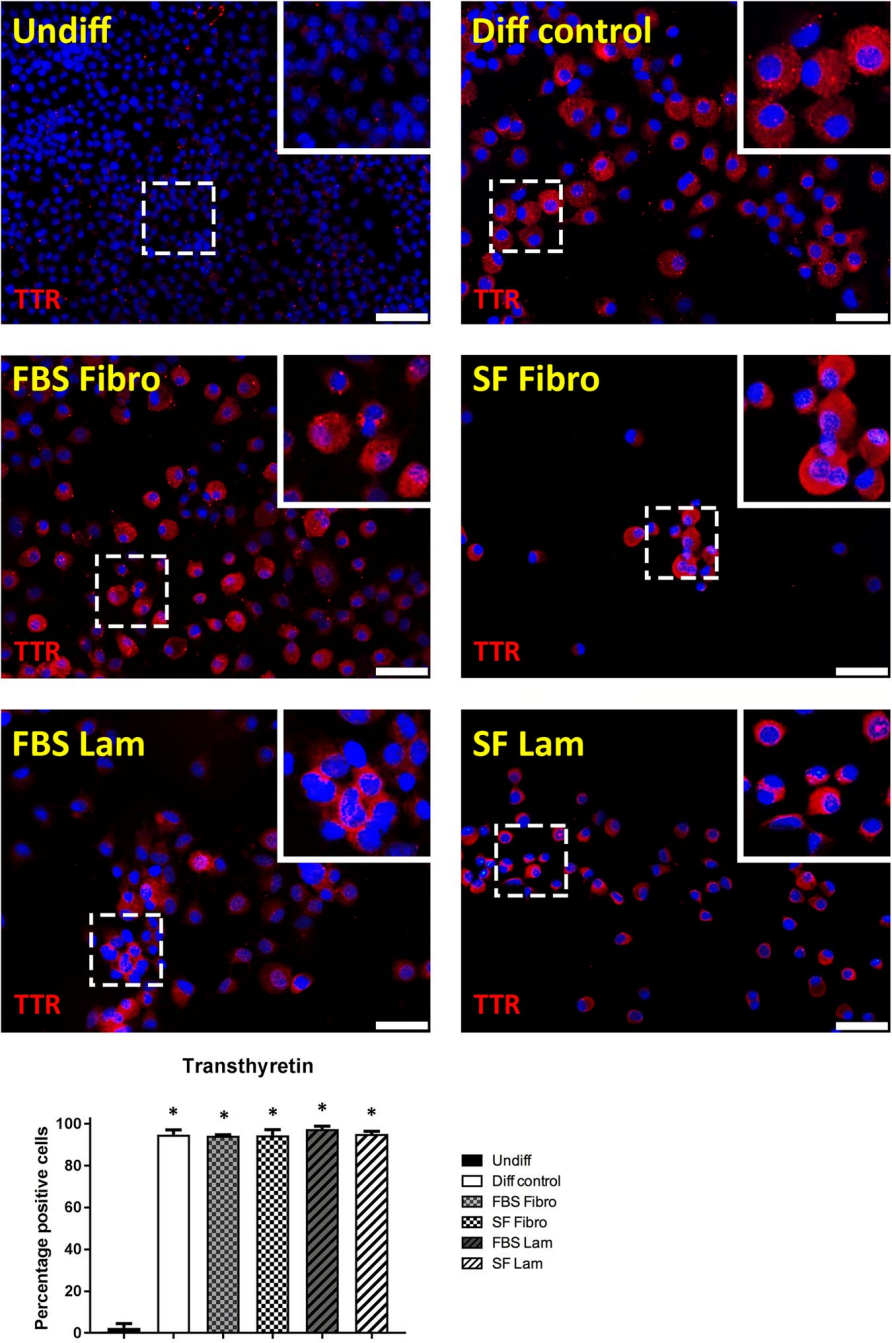
Immunofluorescent staining and quantification of the hepatocyte marker transthyretin (red) in undifferentiated AR42J-B13 cells and cells subjected to differentiation-inducing conditions on day 5, with DAPI (blue) for nuclear quantitation. Undiff - undifferentiated cells; Diff control - differentiation control, with FBS, on plastic; FBS Fibro - differentiation medium, with FBS, on fibronectin; SF Fibro - differentiation medium, serum-free, on fibronectin; FBS Lam - differentiation medium, with FBS, on laminin; SF Lam - differentiation medium, serum-free, on laminin. Scale bar = 50 µm.

To examine the potentially induced pancreas-to-hepatocyte switch at the protein level, we immunofluorescently stained for pancreatic amylase and HNF4α, a nuclear receptor critical for hepatocyte differentiation, in undifferentiated cells versus AR42J-B13 cells subjected to transdifferentation conditions for five days. As expected, amylase was reliably detected at varying degrees in the cytoplasm of 99.5 ± 0.6% of cells grown under basal culture conditions (Fig. 4, top left). However interestingly, exposure to differentiation medium led to downregulation of amylase in a subpopulation of cells while other cells stained robustly. Double staining with antibodies recognising HNF4α or amylase revealed that undifferentiated AR42J-B13 cells did not express this nuclear receptor, whilst it was detected in the majority (61.4 ± 12.6%) of all cells subjected to differentiation-inducing conditions, with some cells co-expressing amylase (suggesting an intermediate phenotype, Fig. 4). To confirm the hepatocyte phenotype of transdifferentiated AR42J-B13 cells, we immuno-stained cells for the serum transport protein transthyretin, which is synthesised and secreted by adult hepatocytes. Undifferentiated, pancreatic AR42J-B13 cells lacked transthyretin expression. In contrast, it was strongly induced in 95.7 ± 1.5% of cells exposed to differentiation medium and the cytoplasm stained in a punctate manner, irrespective of the ECM protein used or whether cells grown on ECM had access to serum (Fig. 5). In addition, all experimental groups were investigated for expression of the perivenous hepatocyte marker glutamine synthetase (GS). Undifferentiated cells were uniformly GS-negative, whereas GS was expressed in a proportion of cells in all experimental groups cultured under hepatocyte differentiation conditions (Supplemental Fig. 1). These data are consistent with results obtained by Tosh and colleagues in a previous study, where treatment of AR42J-B13 cells with Dexamethasone induced the differentiation into perivenous GS^+^ and periportal, carbamoylphosphate synthetase I-expressing hepatocytes^14^.

### Transdifferentiated AR42J-B13 cells display hepatocyte functionality

**Figure 6:**
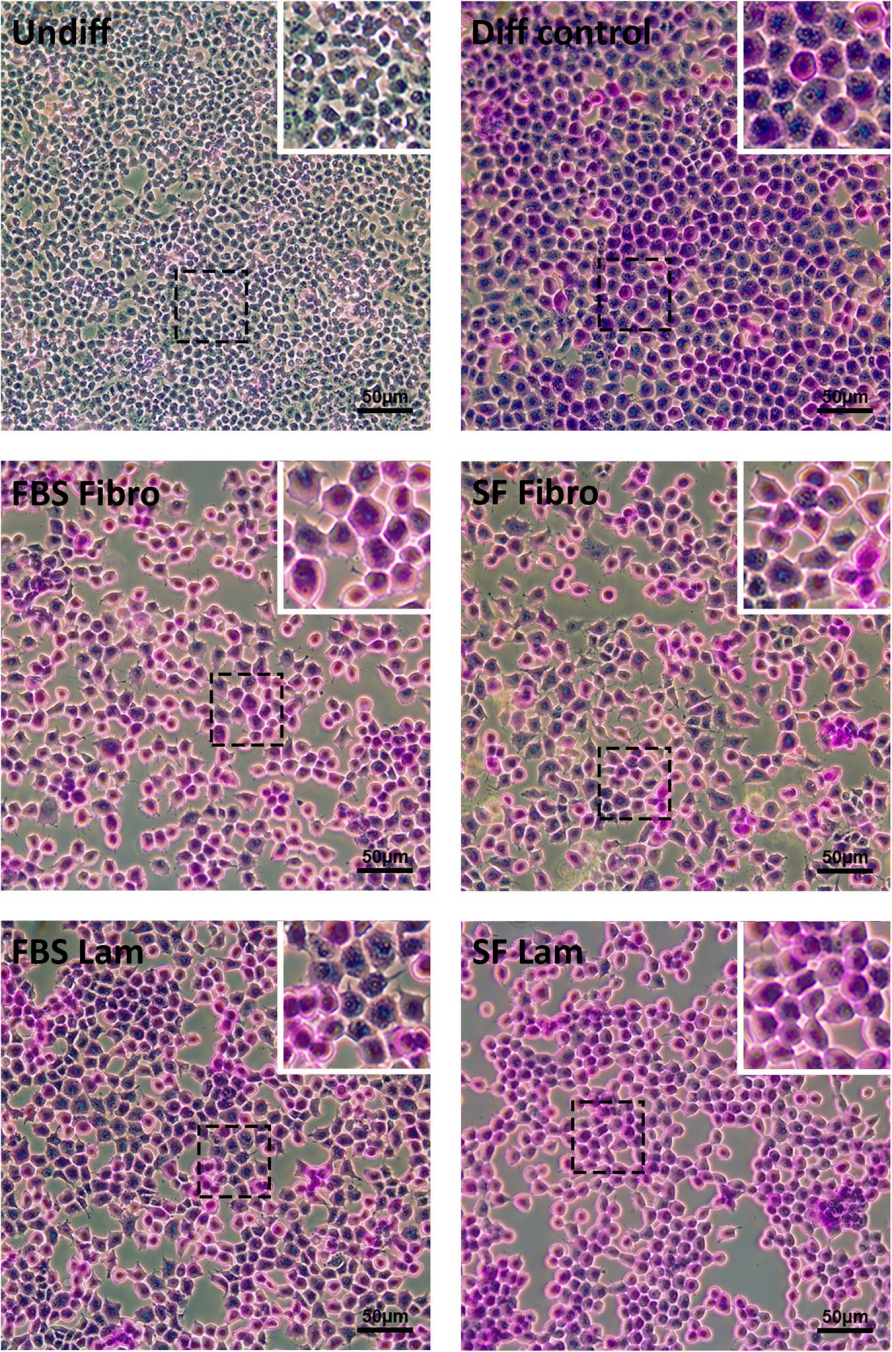
Glycogen storage potential in undifferentiated AR42J-B13 cells and cells subjected to differentiation-inducing conditions on day 5, assessed by Periodic acid-Schiff staining. Undiff - undifferentiated cells; Diff control - differentiation control, with FBS, on plastic; FBS Fibro - differentiation medium, with FBS, on fibronectin; SF Fibro - differentiation medium, serum-free, on fibronectin; FBS Lam - differentiation medium, with FBS, on laminin; SF Lam - differentiation medium, serum-free, on laminin. Scale bar = 50 µm.

**Figure 7:**
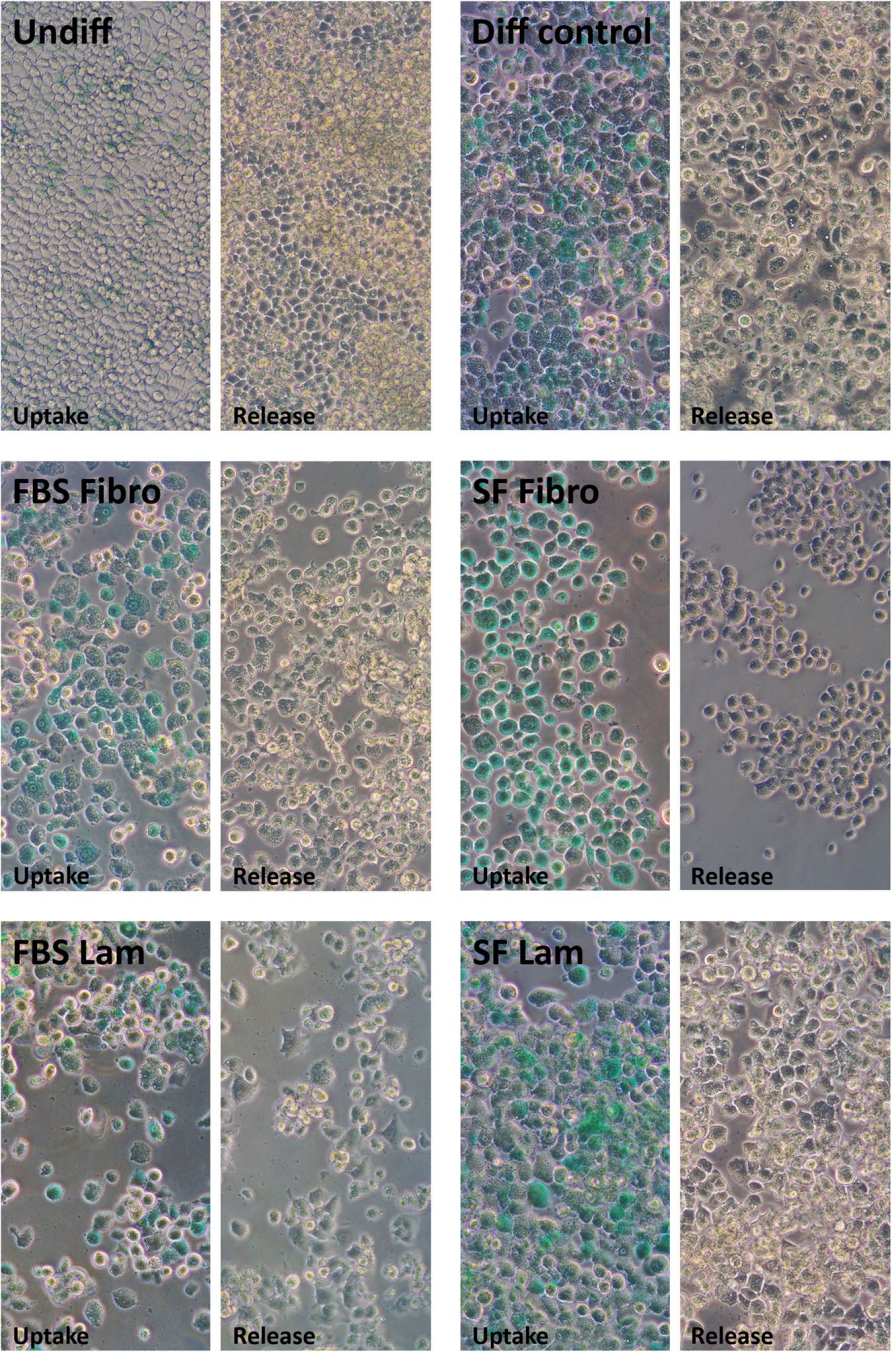
Indocyanine green uptake and release in undifferentiated AR42J-B13 cells and cells subjected to differentiation-inducing conditions on day 5, as a measure of hepatocyte functionality. Undiff - undifferentiated cells; Diff control - differentiation control, with FBS, on plastic; FBS Fibro - differentiation medium, with FBS, on fibronectin; SF Fibro - differentiation medium, serum-free, on fibronectin; FBS Lam - differentiation medium, with FBS, on laminin; SF Lam - differentiation medium, serum-free, on laminin.

**Figure 8:**
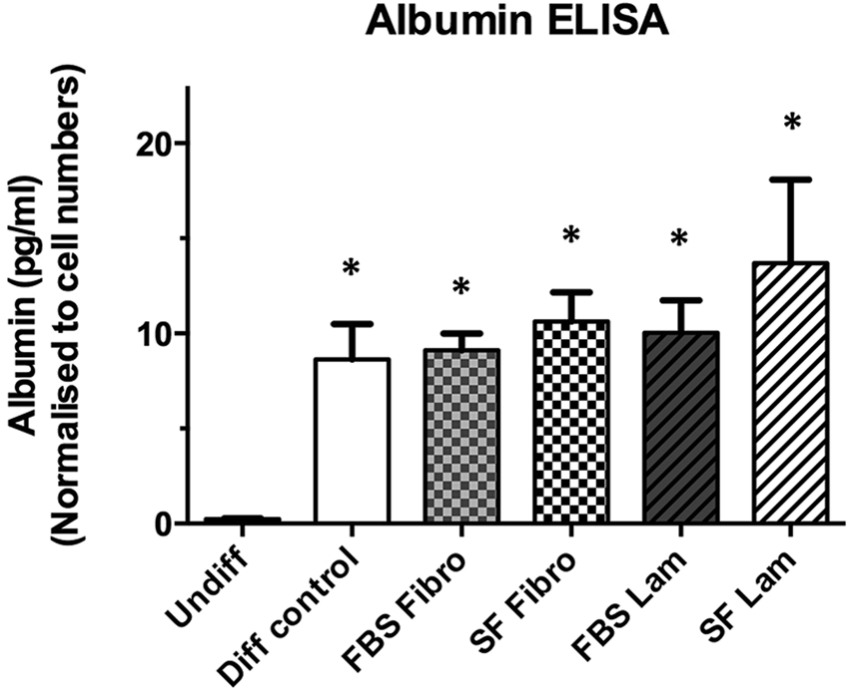
Albumin secretion of undifferentiated AR42J-B13 cells and cells subjected to differentiation-inducing conditions on day 5, measured by ELISA-based assay. Undiff - undifferentiated cells; Diff control - differentiation control, with FBS, on plastic; FBS Fibro - differentiation medium, with FBS, on fibronectin; SF Fibro - differentiation medium, serum-free, on fibronectin; FBS Lam - differentiation medium, with FBS, on laminin; SF Lam - differentiation medium, serum-free, on laminin. The data are expressed as means ± SEM with *n* = 3 and *p < 0.05 compared with undifferentiated cells.

To determine if the transdifferentiated AR42J-B13 cells represented functional HLCs, we utilised multiple assays. Firstly, a Periodic acid-Schiff staining procedure was performed to evaluate the glycogen storage potential of cells in the different experimental groups. Undifferentiated cells did not display any intracellular purple stain and only dispersed, light purple background staining was observed extracellularly (Fig. 6, top left). In contrast, in all other experimental conditions, glycogen was visualised through strong intracellular purple staining in the vast majority of cells (Fig. 6), suggesting functional HLCs. These findings were corroborated using the clinically relevant indocyanine green assay, which is based on the exclusive basolateral uptake and release of a water-soluble, green tricarbocyanine dye by functional hepatocytes. As expected, undifferentiated pancreatic AR42J-B13 cells did not take up any indocyanine green and only faint, extracellular, background staining was observed for this group due to non-specific binding of the dye to some cells (Fig. 7, top left). However in all other experimental groups the cellular uptake of indocyanine green was followed by a steady secretion with complete clearance after about six hours, independent of the ECM protein used or the availability of serum in the substratum-coated dishes (Fig. 7). Lastly, an albumin ELISA was performed to assess hepatocyte functionality. The cell culture supernatant of undifferentiated AR42J-B13 cells did not contain any secreted albumin (of rat origin), whereas albumin secretion was clearly demonstrated for all other experimental groups (Fig. 8).

## Discussion

As a progression from earlier reports using the AR42J-B13 cell line as a pancreas-to-liver transdifferentiation model^5–7,14^, we demonstrate in this study that these pancreatic progenitor cells can reliably be induced to transdifferentiate into functional HLCs. Importantly, this is the first report showing that AR42J-B13 cells readily convert to a hepatocyte phenotype without the use of serum in the culture medium when grown on either fibronectin- or laminin-coated culture substrata.

Although the process of pancreas-to-liver transdifferentiation was originally discussed controversially, it is now well accepted that this can occur *in vivo* and *in vitro* under appropriate conditions^9,24^, but the underlying mechanisms are still unclear. It was shown that extended culture of AR42J-B13 cells with the corticosteroid Dexamethasone induces their hepatocytic conversion through glucocorticoid receptor engagement and downstream activation of CCAAT enhancer binding protein β (C/EBPβ), followed by HNF4α translocation to the nucleus, which in turn activates target genes that mediate the switch to a hepatocytic phenotype^7^. These data were recently extended by Fairhall *et al*. who demonstrated that exposure of AR42J-B13 cells to Dexamethasone resulted in a glucocorticoid receptor-dependent pulse in irreversible DNA methylation and histone modifications. These epigenetic changes then led to constitutive expression of serine/threonine protein kinase 1 isoforms that cross-talk to the Wnt signalling pathway, causing a transient repression in Wnt activity, coupled with the induction of the pancreas-to-liver master regulator C/EBPβ^6^.

To induce and characterise the conversion of AR42J-B13 cells to HLCs under serum-free conditions, these cells were subjected to differentiation-inducing conditions while grown on an ECM glycoprotein. We then assessed them morphologically, at the RNA and protein level and also performed hepatocyte function assays. Our data demonstrate that AR42J-B13 cells readily transdifferentiate into functional HLCs without serum supplementation when seeded on fibronectin or laminin. This conversion was achieved within five days, which is considerably shorter than reported in previous studies, where two to four weeks of Dexamethasone exposure in conjunction with OSM and HGF treatments were necessary for AR42J-B13 cells to transdifferentiate into hepatocyte-like cells^7,14^. Our defined differentiation medium was tailored to mimic the *in vivo* environment in late-stage liver development and hepatic fate specification and was based on serum-free media formulations developed for hepatocyte growth by us and others^14,25–27^. It included Dexamethasone, OSM, HGF as well as FGF-2 and other hepatocyte maturation- and survival-promoting factors such as insulin, transferrin, selenium and nicotinamide^27–29^.

The effect of coating the substratum with defined quantities of either fibronectin or laminin to mimic aspects of the liver microenvironment during the differentiation process was also assessed. Both ECM proteins have been shown to influence the behaviour of liver progenitor cells, which are recruited to regenerate the liver when the hepatocyte pool is chronically injured or inhibited through replicative arrest^23,30^. During chronic liver injury, liver progenitor cells are activated and proliferate in an environment rich in laminin. Laminin is mainly provided by the basement membrane at the basolateral surface of endothelial and epithelial cells^21,31–33^ or by hepatic stellate cells that are in close spatial contact with migrating liver progenitor cells^34–36^. In cell culture experiments, Lorenzini and colleagues demonstrated that growth on laminin kept liver progenitor cells in an undifferentiated state, whereas fibronectin induced hepatocytic differentiation through upregulation of the key hepatic transcription factor C/EBPα^21^. As laminin and fibronectin were shown to significantly impact the differentiation potential of liver progenitor cells, this prompted our choice of these ECM glycoproteins for the pancreas-to-liver transdifferentiation experiments.

In our hands no difference was observed between laminin and fibronectin with regards to their influence on the hepatocytic transdifferentiation potential of AR42J-B13 cells. By day 5, cells in both experimental groups had drastically changed their morphology and their molecular phenotype resembled hepatocytes. The increased amylase expression, which is typical for AR42J-B13 pancreas-to-liver transdifferentiation induced by Dexamethasone^5,37^, and the significantly downregulated PTF1A and pancreatic lipase levels with a concomitant increase in the hepatocytic markers HNF4α, albumin and TAT are all conducive with transdifferentiation. Immunofluorescent analyses of amylase, HNF4α and transthyretin levels confirmed the hepatocytic conversion at the protein level. Lastly, data from two hepatocyte function assays demonstrated that our serum-free culture protocol successfully induced a functional hepatocyte phenotype in AR42J-B13 cells after only five days. This was achieved through defined culture conditions in conjunction with substrata coatings of either of the ECM glycoproteins, fibronectin or laminin.

Key future directions will revolve around the long-term growth and characterisation of transdifferentiated AR42J-B13 cells to elucidate how their hepatocytic phenotype is best maintained in culture. In addition, it would be desirable to establish a human counterpart to the AR42J-B13 cells as a pancreas-to-liver transdifferentiation and hepatocyte precursor model, with the long-term goal of serum-free hepatocyte generation and subsequent cell transplantation in mind.

This study is the first report of successful pancreas-to-liver transdifferentiation of the AR42J-B13 cell line under serum-free conditions, which was achieved after only five days and facilitated by fibronectin or laminin substrata. Further research is required before these results on a rat pancreatic cell line can be translated to produce clinical outcomes. However, the development of a serum-free system on defined extracellular matrices inducing HLCs from pancreatic cells may facilitate the design of substrata that can regulate specific cellular functions in the context of a bioartificial liver system. Our data therefore provide an important stepping-stone towards achieving this long-term goal.

## Materials and Methods

### Growth and transdifferentiation of B13 cells

AR42J-B13 cells were maintained in low glucose Dulbecco’s modified Eagle’s medium (DMEM) + GlutaMAX® (Gibco, Melbourne, Australia), supplemented with 5% FBS (Serana, Bunbury, Australia). Negative control fibroblasts (3T3 cells; ATCC, Manassas, VA, USA) were routinely cultured in RPMI medium (Gibco), supplemented with 10% FBS, 1 mM sodium pyruvate (Hyclone, Laboratories, Logan, UT, USA), and 10 mM 4-(2-hydroxyethyl)-1-piperazineethanesulfonic acid (HEPES, Hyclone), while positive control HepG2 cells (ATCC) were cultured in DMEM (regular glucose), with 10% FBS, 2 mM GlutaMAX® and 10 mM HEPES. All cells were cultured at 37 °C in 5% CO_2_.

‘Basal medium’ used for controls in transdifferentiation experiments consisted of Advanced DMEM/F12 medium (Gibco), supplemented with 0.22 µl/ml monothioglycerol (MTG; Sigma-Aldrich, Castle Hill, Australia), 2 mM GlutaMAX®, and 10 µl/ml HEPES. ‘Differentiation medium’ consisted of basal medium supplemented with 100 nM Dexamethasone (Sigma), 10 ng/ml Oncostatin M (OSM; Sapphire Biosciences, Sydney, Australia), 20 ng/ml hepatocyte growth factor (HGF; Sapphire Biosciences), 20 ng/ml basic fibroblast growth factor (FGF-2; PeproTech, Brisbane, Australia), 1% ITS + (generating a final concentration of 6.25 µg/ml insulin, 6.25 µg/ml transferrin, 6.25 ng/ml selenous acid, 1.25 mg/ml bovine serum albumin, and 5.35 µg/ml linoleic acid; BD Biosciences, North Ryde, Australia), 0.2% additional bovine serum albumin (BSA, Sigma), 4.4 mM nicotinamide (Sigma), 1 µM copper sulphate (Sigma), 30 µg/ml ascorbic acid (Wako Chemicals, Richmond, VA, USA), and 1 µg/ml heparin (Celsus Laboratories, Cincinnati, OH, USA). The media were supplemented with 0 or 5% FBS, as specified.

### Differentiation Assays

Glass coverslips were etched by submersion and mixing in etch solution (6.0 g NaOH dissolved in 24 ml ddH_2_O and the volume made up to 60 ml with 95% ethanol) at room temperature for 30 min, followed by washing with ddH_2_O. After being air-dried and sterilised by UV irradiation for at least 45 min, they were coated with laminin (2 µg/cm^2^; laminin 111 from murine EHS tumours; Sigma-Aldrich) or fibronectin from human plasma (5 µg/cm^2^; Sigma-Aldrich) and used for cell culture in 24-well tissue culture plates (Nunc, Thermo Fisher, Waltham, MA, USA). Replicate wells containing etched coverslips were seeded with 2 × 10^5^ AR42J-B13 cells for RNA extractions and for indirect immunofluorescence detection of protein markers. Experimental groups were as follows: (1) undifferentiated cells in basal medium containing 5% FBS (undiff), (2) differentiation control (diff control): uncoated substratum and cells in differentiation medium with 5% FBS, (3) fibronectin-coated substratum and cells in differentiation medium with 5% FBS (FBS Fibro), (4) fibronectin-coated substratum and cells in differentiation medium (no serum), (SF Fibro), (5) laminin-coated substratum and cells in differentiation medium with 5% FBS (FBS Lam) and (6) laminin-coated substratum and cells in differentiation medium (no serum), (SF Lam). Cells were monitored by phase contrast microscopy and 50% of the medium was replaced daily.

### Quantitative RT-PCR

For each experimental group, replicates (n ≥ 4) grown in 24-well plates (Nunclon, Nunc) were washed gently with PBS and harvested using TRIzol™ RNA isolation reagent (Life Technologies, Melbourne, Australia), according to the manufacturer’s protocol. Eluted RNA was purified and DNase-treated using Isolate II RNA Mini Kit (Bioline, London, UK). After quantitation using a NanoDrop 1000 spectrophotometer (Thermo Fisher), 500 ng of RNA were reverse-transcribed using M-MLV Reverse Transcriptase, RNase H Minus, Point Mutant Kit (Promega, Alexandria, Australia) and random primers. The resulting cDNA was amplified using GoTaq® qPCR Master Mix (Promega) in technical duplicates on a 96-well plate using the ViiA™ 7 Real-Time PCR System (Applied Biosystems, Melbourne, Australia).

Primers used were: rat amylase, forward 5′-TCGATGGCGTCAAATCAGGA-3 and reverse 5′TGTGCCAGCAGCAGGAAGACCAG-3 primers. Rat Qiagen QuantiTect Primer assays used were: hepatocyte nuclear factor 4 α (HNF4α) - QT00188223, tyrosine aminotransferase (TAT) - QT00182308, albumin - QT00189679, pancreas transcription factor 1 subunit alpha (PTF1A) - QT00380527, pancreatic lipase - QT00182371 and 18 S rRNA - QT00199374 (Qiagen, Venlo, Netherlands).

### Immunofluorescence

After five days of growth on etched coverslips, AR42J-B13 cells were rinsed with PBS, fixed with 4% paraformaldehyde (PFA, Sigma) in PBS for 15 min, permeabilised with 1% Triton® X-100 (Merck, Darmstadt, Germany) in PBS for 10 min and blocked with 10% FBS and 1% BSA in PBS (blocking solution) for 30 min. Cells were incubated with primary antibodies in blocking solution for 1 h in a humidified chamber, rinsed with PBS for 10 min, and incubated with secondary antibodies in blocking solution for 1 h. Following a PBS washing step, the coverslips were mounted onto glass slides using ProLong® Gold Antifade Reagent with DAPI nuclear stain (Invitrogen, Melbourne, Australia). For antibodies with cross-reactivity to human epitopes, primary antibody positive control staining was carried out on HepG2 (human liver carcinoma) cells as a hepatocyte model. Cell specificity control staining was performed on 3T3 (mouse fibroblast) cells. Negative controls were omission of the primary antibody, but with secondary antibody present. Primary antibodies: rabbit polyclonal anti-amylase (1:200, Sigma), rabbit polyclonal anti-transthyretin (1:100, Dako, Melbourne, Australia), and mouse monoclonal anti-HNF4α (1:1000, clone K9218, Abcam, Melbourne, Australia). Secondary antibodies: Alexa Fluor® 594 goat anti-mouse (1:400), Alexa Fluor® 488 goat anti-rat (1:400), Alexa Fluor® 488 goat anti-rabbit (1:400), and Alexa Fluor® 594 goat anti-rabbit (1:400), all from Life Technologies, Scoresby, Australia. Glutamine synthetase staining was performed as previously published^14^.

### Periodic Acid-Schiff Assay

AR42J-B13 cells grown for five days on etched coverslips in quadruplicate wells (see section ‘Differentiation Assays’) were rinsed carefully with PBS and fixed with 4% PFA for 15 minutes. Each coverslip was stained using the Periodic Acid-Schiff (PAS) Kit (Sigma), according to the manufacturer’s protocol, modified to a shorter (8 min) Schiff’s reagent incubation and more thorough washing step. Slides were prepared by mounting the coverslips using aqueous glycine jelly mount consisting of 1 g bovine hide gelatine (Davis Gelatine, Botany, Australia), 6 ml distilled water, 7 ml glycerol (VWR, Tingalpa, Australia) and 25 mg phenol (VWR).

### Indocyanine Green Uptake and Release Assay

AR42J-B13 cells grown for five days (see section ‘Differentiation Assays’) were incubated with diluted indocyanine green (Sigma, 1 mg/ml) for 30 min 37 °C in 5% CO_2._ Cultures were washed and imaged to establish the degree of indocyanine green uptake. Cells were cultured for an additional 6 h, washed and imaged again to determine the release of intracellular indocyanine green stain.

### Albumin Enzyme-Linked Immunosorbent Assay

Cell culture supernatants of AR42J-B13 cells grown for five days (see section ‘Differentiation Assays’) were collected and investigated for the presence of secreted albumin using a commercially available rat albumin ELISA kit (ab108789, Abcam), according to the manufacturer’s instructions. Results were normalised to cell numbers.

### Scanning Electron Microscopy

After five days of growth on etched coverslips in duplicate wells, AR42J-B13 cells were rinsed with PBS and immersed in an incremental gradient of 30, 50, 70, 90 and 100% molecular grade ethanol (Sigma), diluted in PBS. Samples were then coated with platinum and visualised with the assistance of Elaine Miller at Curtin University’s Microscopy and Microanalysis Facility on a Zeiss Neon 40EsB FIBSEM (Zeiss Australia, North Ryde, NSW, Australia).

### Live Cell Imaging

AR42J-B13 cells were seeded as per standard differentiation assay in triplicate wells of a 24-well plate for each condition and cultured for four days using the INU stage-top incubation system for inverted Nikon microscopes (Tokai Hit, Fujinomiya, Japan). Images were taken every 30 min in the same X, Y and Z co-ordinates.

### Microscopy

Phase contrast microscopy was conducted using an Axiovert S100 microscope (Zeiss), and immunofluorescent microscopy using an Olympus BX51 microscope (Olympus, Perth, Australia). Live cell imaging microscopy utilised the Nikon A1+ Confocal Laser Microscope (Nikon Instruments Inc., Melville, NY, USA).

### Statistical analysis

Quantitative data from transcriptomic analyses are presented as means ± standard error of the mean (SEM). Statistical significance was assessed by parametric one-way analysis of variance of log-transformed data using Graphpad Prism 6 for Mac OS X (Graphpad Software, Inc., San Diego, CA, USA). Values of p < 0.05 were considered statistically significant.

## Electronic supplementary material

Supplemental online video 1

Supplementary Information
