# Supplementary material for: Transdifferentiation of pancreatic progenitor cells to hepatocyte-like cells is not serum-dependent when facilitated by extracellular matrix proteins
Source: Sci Rep. 2018 Mar 12;8:4385. doi: 10.1038/s41598-018-22596-z (PMC5847606; doi:10.1038/s41598-018-22596-z)
Supplement: Supplementary file 2 — Supplementary Information [file 41598_2018_22596_MOESM2_ESM.pdf]

Transdifferentiation of pancreatic progenitor cells to hepatocyte-like cells is not serum-dependent when facilitated by extracellular matrix proteins

Francis D. Gratte, Sara Pasic, John K. Olynyk, George C. T. Yeoh, David Tosh, Deirdre R. Coombe and Janina E. E. Tirnitz-Parker

Supplemental Figure Legend

Supplemental Online Video 1

Live cell imaging of cells grown under differentiation-inducing conditions on laminin and without FBS for four days.

Supplemental Figure 1

Immunofluorescent staining of glutamine synthetase (red) in undifferentiated AR42J-B13 cells and cells subjected to differentiation-inducing conditions on day 5, with DAPI (blue) for nuclear visualisation. Undiff - undifferentiated cells; Diff control - differentiation control, with FBS, on plastic; FBS Fibro - differentiation medium, with FBS, on fibronectin; SF Fibro - differentiation medium, serum-free, on fibronectin; FBS Lam - differentiation medium, with FBS, on laminin; SF Lam - differentiation medium, serum-free, on laminin.

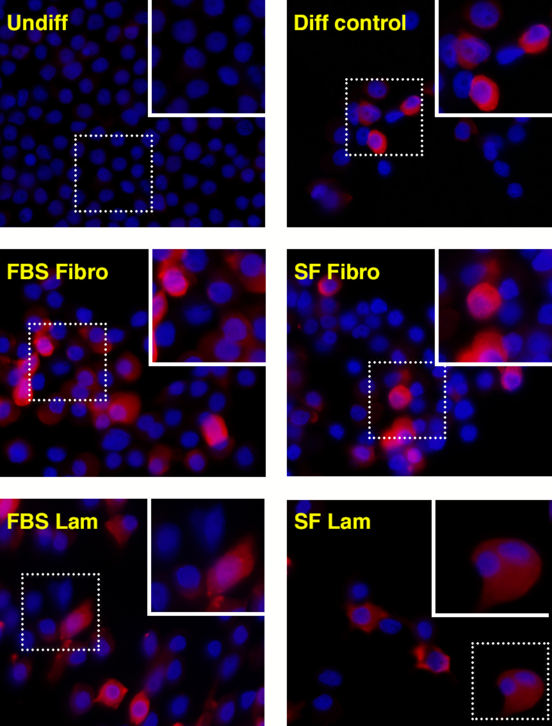
Undiff
Diff control
FBS Fibro
SF Fibro
FBS Lam
SF Lam
